# Orexin-A exerts neuroprotective effect in experimental intracerebral hemorrhage by suppressing autophagy *via* OXR1-mediated ERK/mTOR signaling pathway

**DOI:** 10.3389/fncel.2022.1045034

**Published:** 2022-12-22

**Authors:** Dexin Zhang, Ying Cui, Manman Zhao, Xuecheng Zheng, Chunyan Li, Jingbo Wei, Kaijie Wang, Jianzhong Cui

**Affiliations:** ^1^Department of Surgery, Hebei Medical University, Shijiazhuang, China; ^2^Department of Neurology, Tangshan Gongren Hospital, Tangshan, China; ^3^Department of Histology and Embryology, North China University of Science and Technology, Tangshan, China; ^4^Department of Neurology, Shandong Provincial Hospital Affiliated to Shandong First Medical University, Jinan, China; ^5^Department of Neurosurgery, Tangshan Gongren Hospital, Tangshan, China

**Keywords:** orexin A, autophagy, intracerebral hemorrhage, neuroprotection, orexin receptor

## Abstract

**Background:**

Orexin-A (OXA) is a polypeptide produced in the hypothalamus, which binds to specific receptors and exerts multiple physiological effects. Autophagy plays a vital role in early brain injury (EBI) after intracerebral hemorrhage (ICH). However, the relationship between OXA and autophagy after ICH has not been confirmed.

**Methods:**

In this study, the protective role of OXA was investigated in a model of hemin-induced injury in PC12 cells and blood-injection ICH model in rats, and its potential molecular mechanism was clarified. Neurobehavioral tests, brain water content, and pathologic morphology were assessed after ICH. Cell survival rate was determined using Cell Counting Kit-8 (CCK-8), while apoptosis was detected using flow cytometry. The autophagy protein LC3 that was originally identified as microtubule-associated protein 1 light 3 was evaluated by immunohistochemistry. The ultrastructural changes of cells following ICH were observed by transmission electron microscopy. Western blotting was performed to determine the expression levels of LC3, p62/SQSTM1 (p62), phosphorylated extracellular signal-regulated kinase 1/2 (p-ERK_1/2_), total extracellular signal-regulated kinase 1/2 (t-ERK_1/2_), mammalian target of rapamycin (mTOR), and phosphorylated mammalian target of rapamycin (p-mTOR).

**Results:**

OXA treatment significantly improved neurofunctional outcomes, reduced brain edema, and alleviated neuronal apoptosis. OXA administration upregulated p-mTOR and p62, while it downregulated p-ERK_1/2_ and LC3; this effect was reversed by the orexin receptor 1 (OXR1) antagonist SB-334867.

**Conclusions:**

This study demonstrates that OXA suppresses autophagy *via* the OXR1-mediated ERK/mTOR signaling pathway to exert neuroprotective effects, and it might provide a novel therapeutic approach in patients suffering from ICH.

## 1 Introduction

Intracerebral hemorrhage (ICH) is the leading cause of disability and death all over the world (Appelboom et al., 2012), affecting approximately 2 million individuals per year (Kang and Yao, 2019), and it causes an enormous economic burden on families and society (Benjamin et al., 2017). Brain injury following ICH includes primary injury caused by direct compression of the hematoma and secondary changes caused by various reasons such as inflammation, oxidative stress, mitochondrial dysfunction, and neuronal death (Kim-Han et al., 2006; Mracsko and Veltkamp, 2014; Hu et al., 2016; Zhao et al., 2018), among which the secondary changes is an important factor that affects prognosis of patients with ICH (Zhao et al., 2015). Therefore, prevention and treatment of secondary brain injury (SBI) occurrence and development after ICH is very valuable (Dang et al., 2017).

Autophagy is a basic biological procedure through which eukaryotic cells degrade organelles and misfolded, or damaged proteins to maintain normal cellular function (Boya et al., 2013; Füllgrabe et al., 2013; Kim et al., 2013). Under the circumstances of oxidative stress and inadequate nutrition, autophagy primarily functions as a cell survival adaptation mechanism (Bennett et al., 2010; Wellnitz and Taegtmeyer, 2010; Wirawan et al., 2012). It has been shown that autophagy plays a critical function in many central nervous system (CNS) diseases, including neurodegenerative diseases (Salminen et al., 2013; Gan-Or et al., 2015) and cerebral ischemia (Kotoda et al., 2018; Yu et al., 2019). Previous studies, including those on neonatal hypoxia–ischemia brain injury (Carloni et al., 2008) and subarachnoid hemorrhage (SAH) (Jing et al., 2012), have suggested that autophagy is an important protective process. Whereas, in other studies, autophagy has been shown to cause apoptosis in models of cerebral ischemia (Rami et al., 2008), traumatic brain injury (Cui et al., 2015) and ICH (Duan et al., 2017). Therefore, the role of autophagy in nerve cells may depend on the injury model and specific the cellular context (Li H. et al., 2018; Zhao et al., 2021). As reported in a previous study, the activation of autophagy following ICH plays a critical role in the mechanism of brain tissue damage (Shen et al., 2016).

Orexins (OXs) are polypeptides primarily produced by neurons in the lateral hypothalamus (Barinaga, 1998; Feng et al., 2014). Orexins exist in two forms—orexin A (OXA) and orexin B (OXB), which play a wide range of roles, including feeding, analgesic effects, sleep/wakefulness, sleep-pain interplay, energy metabolism, and drug addiction (Mohammad Ahmadi Soleimani et al., 2015; Ahmadi-Soleimani et al., 2020; Li et al., 2020), by binding to two G-protein-coupled receptors (GPCRs), orexin receptor 1 (OXR1), and orexin receptor 2 (OXR2) (Wang et al., 2018). Although the functions of OXs are diverse, one of the main functions of OXs is protection, particularly in neurological disorders. It has been reported that exogenous OXA administration has a neuroprotective effect on dopaminergic neurons in Parkinson’s disease (Drouot et al., 2003). Other studies on rat model of cerebral ischemia have shown similar neuroprotective effects (Kitamura et al., 2010).

Research has confirmed the relationship between the protective role of OXA and autophagy in models of cerebral ischemia–reperfusion injury (CIRI) or middle cerebral artery occlusion (MCAO). However, relatively few studies have assessed the relationship between OXA and autophagy in ICH. In this research, we hypothesized that OXA could exert a protective role on ICH-induced brain injury through inhibiting autophagy. Subsequently, the *in vivo* and *in vitro* model of ICH was developed, and a variety of experiments were conducted to verify our hypothesis and to provide new ideas for the treatment strategy of ICH.

## 2 Materials and methods

### 2.1 The animals

A total of 100 healthy adult male Sprague-Dawley (SD) rats (250–300 g, 10–12 weeks) were purchased from BEIJING HUAFUKANG BIOSCIENCE Co., Ltd. (Beijing, China) and housed in a standard environment (temperature of 25°C, humidity of 55%, and 12 h light/dark cycles) with free access food and water. All experimental schemes were sanctioned by the Laboratory Animal Ethical and Welfare Committee of North China University of Science and Technology (approval number: SQ 2022170) and followed the National Institutes of Health (NIH) Guide on Laboratory Animal Care and Use. People strictly follow the “3R” principle (Replace, Reduce, Refine) to carry out this operation.

### 2.2 Cell culture

The PC12 cell line of the rat adrenal medulla pheochromocytoma, purchased from the Cell Resource Center of the Chinese Academy of Sciences (Shanghai, China), were cultured in Dulbecco’s modified Eagle’s medium (Gibco; CA, USA) with 10% fetal bovine serum (Bovogen; Melbourne, VIC, AUS) and 1% penicillin/streptomycin antibiotics (Gibco; CA, USA) at 37°C in a humid environment containing 5% CO_2_ and 95% air. After being grown to the appropriate concentration, the cells were seeded into 96-well plates, 60 mm dishes, and 100 mm dishes for subsequent experiments.

### 2.3 Intracerebral hemorrhage (ICH) models

#### 2.3.1 ICH model *in vitro*

As previously described (Zhou et al., 2017), an *in vitro* model of ICH was established by treating PC12 cells with hemin. Briefly, when PC12 cells were normally cultured to 70–80% confluency, they were stimulated with hemin for a period of time for construction of the *in vitro* model of ICH.

#### 2.3.2 ICH model *in vivo*

According to the previous study (Yang et al., 2021), autologous blood from the tail artery was injected into the right basal ganglia to establish an *in vivo* ICH model. Briefly, an intraperitoneal injection of 10% chloral hydrate was used to anesthetize the animals, and then, they were fixed by a stereotaxic instrument (Stoelting; Kiel, WI, USA). A total of 50 μL autologous blood from the tail artery of the rat was infused into the right basal ganglia *via* a 26-gauge needle at a speed of 3.33 μL/min using a micro-infusion pump (Harvard Apparatus Inc., South Natick, MA, USA) at the following coordinates relative to bregma: 0.2 mm anterior, 3.5 mm right lateral, and 5.5 mm deep. Rats in the sham group only received a needle insertion without injection. In order to prevent backflow, the needle remained in place for an additional 5 min after intracerebral infusion. ICH rats were received intracerebroventricular injection (2 μL/min) of OXA drug solution (30 μg/kg, Phoenix Pharmaceuticals; Belmont, CA, USA) or vehicle (Dimethyl sulfoxide) 1 h after ICH induction (coordinates: 0.8 mm posterior and 1.6 mm lateral of bregma and at a depth of 4.0 mm).

### 2.4 Study design

#### 2.4.1 Experiment 1: Time-course changes of LC3 and p62

Twenty-seven rats (30 rats suffered to the surgery, 27 rats survived) were randomly and evenly allotted to 9 groups of 3 rats each, namely, sham group, ICH 12 h group, ICH 24 h group, ICH 48 h group, ICH 3 d group, ICH 5 d group, ICH 7 d group, ICH 14 d group, ICH 28 d group. The expression of autophagy-related proteins (including LC3B and p62) in the perihematomal tissue was detected by Western blot (WB) analysis.

#### 2.4.2 Experiment 2: Evaluate protective effects of OXA

Sixty-four rats (70 rats experienced the operation, 64 rats survived) were randomly assigned into 4 groups (16 rats per group): sham group, ICH group, ICH + vehicle (dimethylsulfoxide, DMSO) group, ICH + OXA 30 μg/kg group. Modified Neurological Severity Score (mNSS) and corner turn test (*n* = 7) were performed at 48 h after ICH to evaluate the neurofunction. After the tests, rat brains were harvested for brain water content (BWC) measurements (*n* = 7). Then, the other rats were taken to perform H&E, Nissl and immunohistochemistry (IHC) staining (*n* = 3), transmission electron microscopy (*n* = 3) and WB (*n* = 3). All rats were sacrificed by deeply anesthetized with an i.p. of 10% chloral hydrate, and then were decapitated. The animal carcasses were frozen and returned to the laboratory animal center for unified harmless treatment.

#### 2.4.3 Experiment 3: Mechanisms of the protective effect of OXA

This part of the experiment was carried out at the cellular level. The cells were randomly divided into seven groups: Control (untreated) group, Hemin group, Hemin + vehicle (DMSO) group, Hemin + OXA (100 nM) group, Hemin + PD98059 group, Hemin + OXA (100 nM) + Rapamycin group, Hemin + OXA (100 nM) + SB334867 group. All groups in this experiment were taken to perform Cell Counting Kit-8 (CCK-8), Western blotting and flow cytometric apoptosis assay to confirm the mechanisms of the protective effect of OXA. All assays were repeated at least three times.

### 2.5 Evaluation of neurological dysfunction

Neurological deficits in rats at 2 days following ICH were evaluated using the modified neurologic severity score (mNSS). The severity score is graded on a scale of 0–18, in which 13–18 indicates severe injury; 7–12 represents moderate injury; and 1–6 expresses mild injury. The higher the score, the more severe the injury. In addition, the short-term neurological outcomes after ICH were also assessed by the corner turn test, as previously described (Xi et al., 2018). The results are expressed as the percentage of left turns to all turns. All tests were assessed by two trained investigators without knowing of the groups.

### 2.6 Brain water content measurement

Brain water content was determined using the wet/dry method, as previously described (Manaenko et al., 2011). In brief, at 48 h after ICH, the rats were decapitated after being deeply anesthetized. Brains were completely removed and weighed immediately to obtain wet weight, and they were desiccated at 105°C for 48 h to acquire dry weight. The percentage of BWC was determined as (wet weight-dry weight)/wet weight × 100%.

### 2.7 H&E and Nissl staining

After being deeply anesthetized, rats were sequentially injected with physiological saline and 4% paraformaldehyde through the heart. The brain tissues were isolated and fixed in 4% paraformaldehyde for 48 h, dehydrated with gradient alcohol, soaked in wax, embedded in paraffin, and then cut into 5 μm thick coronal section. H&E and Nissl staining were performed as previously described (Ning et al., 2019). The slides were mounted using neutral gum and observed under an optical microscope (BX51, Olympus, Tokyo, Japan).

### 2.8 Immunohistochemistry

Briefly, 5 μm thick brain tissue sections were prepared, as described above. Immunohistochemical staining was carried out in accordance with the instructions of immunohistochemistry kit (ZSJQ-BIO, Beijing, China). In brief, brain tissue slices were dewaxed and hydrated, and then citrate buffer was added to perform heat-induced antigen retrieval for 10 min, and then cooled to room temperature naturally. The slices were treated with 3% hydrogen peroxide to inactivate endogenous peroxidase at 25°C for 5 min. Rabbit anti-microtubule-associated protein light chain-3 polyclonal antibodies (GeneTex; Irvine, CA, USA; 1:50 dilution) were added, and then incubated overnight at 4°C; and subsequently incubated with horseradish peroxidase- (HRP) conjugated second antibody for 60 min, and then colored by diaminobenzidine (DAB). The sections were observed under an optical microscope at 400 × magnification and photographed.

### 2.9 Transmission electron microscopy

After anesthesia, rats were transcardially injected with saline, and then replaced with 4% paraformaldehyde and 2.5% glutaraldehyde. The brain tissue was completely removed and fixed in 2.5% glutaraldehyde at 4°C for 24 h, and then fixed with 1% osmium tetroxide for 90 min. After dehydration with graded alcohol, the tissues were permeated with propylene oxide and embedded in epoxy resin. Ultrathin sections were prepared and stained with 4% uranyl acetate for 20 min, and then stained with lead citrate for 5 min. The sections were observed using transmission electron microscopy (Hitachi, Tokyo, Japan).

### 2.10 Cell-viability assay

According to the manufacturer’s instructions, the CCK-8 kit was used to determine the viability of PC12 cells. PC12 cells were seeded into 96-well plates at an initial density of 1 × 10^4^ per well and cultured for 24 h. The intervention of cells was performed according to the experimental requirements. After intervention, a total of 10 μL volume CCK-8 reagent was added each well and incubated for 1 h. A microplate reader was used to measure the optical density (OD value) of each well at 450 nm wavelength. All assays were repeated at least three times. Cell viability was calculated using the following formula: cell viability (%) = (OD value of experimental group/average OD value of control group) × 100%.

### 2.11 Flow cytometric analysis of apoptosis

In accordance with the manufacturer’s instructions, flow cytometry was used to detect apoptosis in PC12 cells after staining with the Annexin V-FITC apoptosis detection kit (Beyotime, Shanghai, China). PC12 cells were planted into 60 mm dishes at an initial density of 2 × 10^5^ and normally cultured to 80–90% confluency. After different treatments, the cells were collected and washed with PBS. Thereafter, the cells were resuspended in 195 μL 1 × binding buffer at a density of 1 × 10^5^/ml and stained with 5 μL Annexin V-FITC and 10 μL propidium iodide for 15 min at 25°C in the dark. After reaction, the treated cells were analyzed by flow cytometer (Beckman Coulter, USA) within 1 h. All assays were repeated at least three times.

### 2.12 Western blot analysis

After anesthesia, the rats were decapitated and the perihematomal brain tissues were gathered. The perihematomal brain tissues and PC12 cells were lysed for 30 min in precooled RIPA lysis buffer (Beyotime, Shanghai, China) including proteinase and phosphatase inhibitors. After centrifugation at 12,000 rpm for 15 min at 4°C, the protein concentration in supernatant was determined by the BCA protein assay kit (Beyotime, Shanghai, China). Equal amounts of protein (50μg) were separated by sodium dodecyl sulfate-polyacrylamide gel electrophoresis (SDS-PAGE) in 6, 10, or 12% polyacrylamide gels and transferred onto 0.22 μm Hybond-polyvinylidene difluoride (PVDF) membranes. The membranes were blocked with 5% non-fat milk in Tris-buffered saline containing 0.05% Tween 20 (TBST, pH 7.4) for 90 min, and then incubated overnight at 4°C with the appropriately diluted primary antibodies: rabbit anti-p-ERK_1/2_ (1:1,000 dilution; HuaAn Biotechnology Co., Ltd., Hangzhou, China), rabbit anti-t-ERK_1/2_ (1:1,000 dilution; HuaAn Biotechnology Co., Ltd., Hangzhou, China), rabbit anti-p-mTOR (1:1,000 dilution; Arigo; Hsinchu, Taiwan, China), rabbit anti-mTOR (1:1,000 dilution; HuaAn Biotechnology Co., Ltd., Hangzhou, China), rabbit anti-LC3B (1:1,000 dilution; GeneTex; Irvine, CA, USA), rabbit anti-p62 (1:1,000 dilution; MBL; Nagoya, Japan), mouse anti-GAPDH (1:1,000 dilution; ABclonal; Wuhan, China), and rabbit anti-β-actin (1:100,000 dilution; ABclonal; Wuhan, China). After washing, the membranes were incubated with the corresponding HRP-conjugated secondary antibodies (1:5,000 dilution; ABclonal; Wuhan, China) for 120 min at 25°C. Blots were visualized with an enhanced chemiluminescence detection kit (ECL; Beyotime, Shanghai, China) and quantified with Image J software.

### 2.13 Statistical analysis

All data are expressed as mean ± standard deviation (mean ± SD). SPSS 25.0 statistical software was used for data analysis, and GraphPad Prism 8.0 software was used to make statistical charts. The differences among multiple groups were determined with one-way ANOVA analysis, followed by *post hoc* Tukey test, and the comparison between two groups was performed by independent samples *t*-test. For all comparisons, *P* < 0.05 was considered statistically significant.

## 3 Results

### 3.1 OXA attenuates secondary brain injury (SBI) after ICH *in vivo*

In order to elucidate the protective effect of OXA, autologous blood injection was performed to establish an ICH model *in vivo*. To evaluate the effect of OXA treatment on early brain injury (EBI) at 48 h after ICH, mNSS and corner turn test were used to assess neurological dysfunctions, and the wet/dry method was used to determine BWC, and H&E and Nissl staining were used to evaluate brain damage.

**FIGURE 1:**
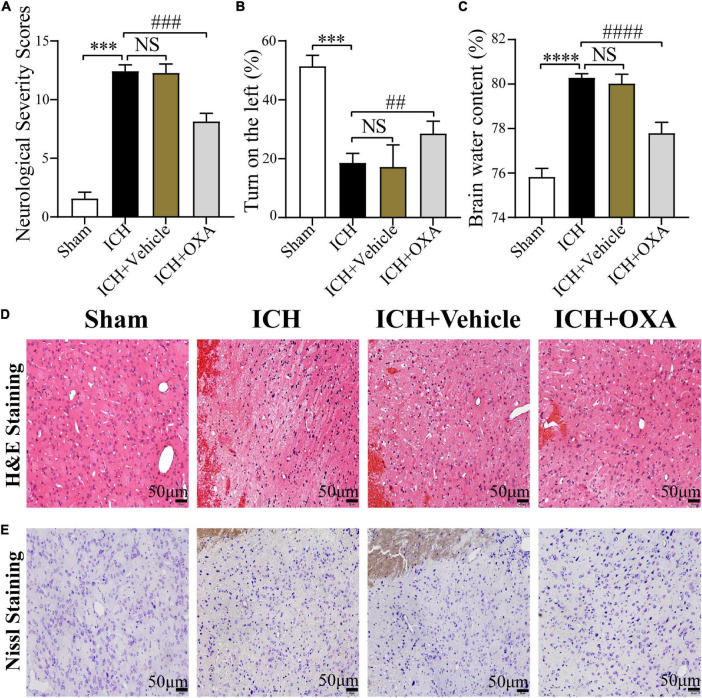
Orexin-A alleviates early brain injury following ICH *in vivo*. **(A)** Modified neurological severity scores (mNSS) and **(B)** corner turn test showed that neurological function was significantly impaired after ICH, and OXA treatment significantly improved outcomes. **(C)** Water content of brain tissues in each group. ^***^p < 0.001 and ^****^p < 0.0001 vs. the sham group; NS, non-significant different compared to the ICH group. ^##^*p* < 0.01, ^###^*p* < 0.001 and ^####^*p* < 0.0001 vs. the ICH group. **(D,E)** Representative images of H&E and Nissl staining in the perihematomal brain tissues region. Scale bar, 50 μm.

The findings showed that the mNSS scores were significantly increased in the 48 h post-ICH and vehicle groups compared with the sham group. There was a significant difference between the OXA and vehicle groups, suggesting that OXA had a positive effect in reducing neurologic impairments (Figure 1A). In the corner turn test, the percentage of left turns in the ICH and vehicle groups was significantly lower than in the sham group, while the percentage of left turns in the OXA treatment group was significantly higher than in the ICH-injury group (Figure 1B). Additionally, the ICH and vehicle groups showed significant increases in the BWC, which was attenuated by OXA treatment (Figure 1C). The results of H&E and Nissl staining revealed that relative to the sham group, neurons in the 48 h post-ICH and vehicle group had a disordered arrangement showing karyopyknosis and cytoplasmic loosening, and the nucleolus had disappeared. In contrast, treatment with OXA alleviate the neuronal damage after ICH (Figures 1D, E). Taken together, these data indicate that OXA has neuroprotective effects after ICH.

### 3.2 Autophagy is activated after ICH

**FIGURE 2:**
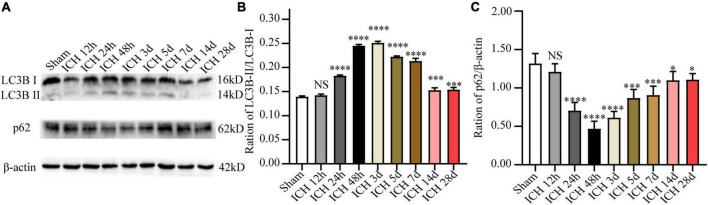
The expression changes of autophagy-related proteins (LC3B and p62) at different time points after ICH. **(A)** Representative western blot images of LC3B and p62. **(B,C)** Quantitative analysis of LC3B and p62 in each group. *p < 0.05, ^***^p < 0.001 and ^****^p < 0.0001 vs. the sham group; NS, non-significant different compared to the sham group.

In order to observe autophagy in the ICH injury model *in vivo*, the expression of autophagy-related proteins (including LC3B and p62) in the perihematomal tissue at different time points after ICH was detected by WB analysis. The results demonstrated that relative to the sham group, the ratio of LC3B-II/LC3B-I after ICH was markedly increased at 12 h, and it peaked at day 2, and decreased markedly at day 14. In contrast, the protein expression level of p62 showed an opposite trend at the same time points (Figure 2). Based on these results, 48 h after ICH were considered the optimal time point for subsequent experiments.

### 3.3 OXA inhibits activation of autophagy

A previous study demonstrated that inhibition of autophagy can reduce brain damage after ICH (Yuan et al., 2017). After verifying that ICH can activate autophagy and OXA has a neuroprotective role in ICH injury, we speculated that OXA may play a neuroprotective role by regulating autophagy activity. A variety of methods were used to monitor autophagy, such as immunohistochemistry, Western blotting etc.

**FIGURE 3:**
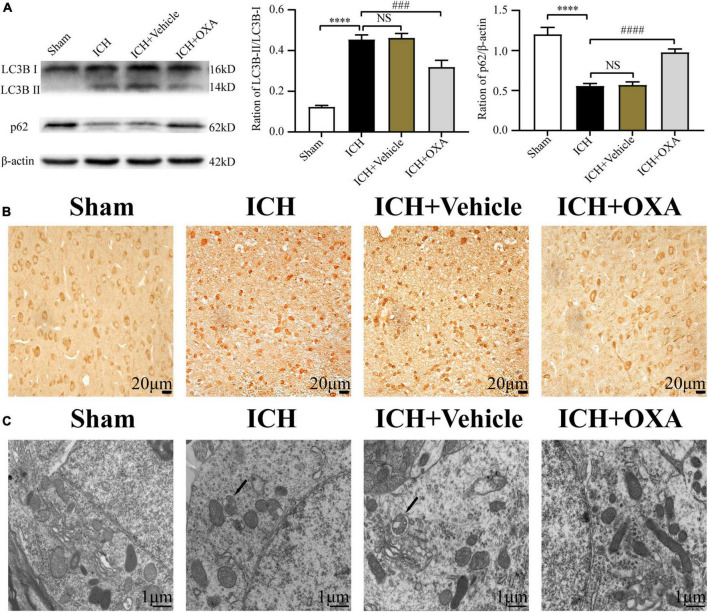
Orexin-A inhibits ICH-induced autophagy activation. **(A)** Western blot images of LC3B and p62 and results of quantitative analysis in the perihematomal area of rats after ICH. **(B)** Representative immunohistochemical staining images of LC3B in each group. Positive cells were stained brown. Scale bar, 20 μm. **(C)** Ultrastructural observation of perihematomal brain tissue after ICH by transmission electron microscopy, black arrows represent autophagosome. Scale bar, 1 μm. ^****^p < 0.0001 vs. the sham group; NS, non-significant different compared to the sham group; ^###^*p* < 0.001, ^####^*p* < 0.0001 vs. the ICH group.

To identify whether OXA could inhabit autophagy *in vivo*, we used Western blotting to analyze the expression of autophagy-related proteins in the perihematomal region after ICH. As shown in Figure 3A, ICH upregulated the ratio of LC3B-II/LC3B-I and downregulated the level of p62. On the contrary, administration of OXA significantly decreased the ratio of LC3B-II/LC3B-I and increased the p62 level. Consist with the Western blotting results, the immunohistochemistry staining had similar trends (Figure 3B). In addition, electron microscopy is believed to be the most sensitive and precise method of monitoring autophagy, which distinctly reveals the presence of autophagosomes and autolysosomes in the area around the hematoma after ICH. In contrast, cells in the same area of sham rats contained few vacuoles, and intact nuclei and mitochondrial crista were observed (Figure 3C). After administration of OXA, we observed that the double-membrane structures were decreased and swollen mitochondria were scattered throughout the cytoplasm, which indicates alleviation of cellular damage.

### 3.4 The neuroprotective effect of OXA is mediated by inhibition of autophagy *via* the ERK/mTOR signaling pathway

In order to verify the above experimental results and elucidate the molecular mechanism of OXA’s neuroprotective effect, we further explored at the cellular level. We used PC12 cells to simulate neurons *in vitro* to exclude the interference between various types of cells in the brain tissue *in vivo* and adopted hemin-induced injury of PC12 cells as an *in vitro* model of ICH.

#### 3.4.1 OXA relieves hemin-induced injury of PC12 cells *in vitro*

**FIGURE 4:**
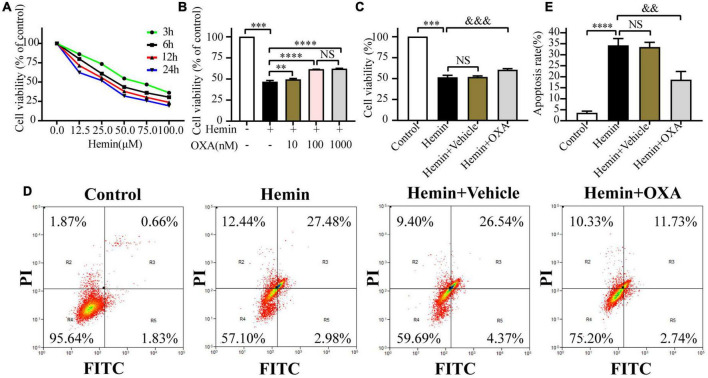
Orexin-A relieves hemin-induced PC12 cells injury *in vitro*. **(A)** Survival curve of hemin-induced PC12 cells injury. **(B)** Effects of treatment with various concentrations of OXA on the hemin-induced decrease in the viability of PC12 cells. ^***^p < 0.001 vs. non-treated group; ^**^p < 0.01 and ^****^p < 0.0001 vs. hemin-treated group; NS, no- significant difference between the two groups. **(C)** OXA attenuates the hemin-induced decrease in cell viability. **(D)** OXA inhibited hemin-induced apoptosis. PC12 cells were divided into four groups, the effects of OXA on apoptosis were detected by flow cytometric, and representative FCM plots are shown. **(E)** Quantified results of the flow cytometric analysis. ^***^p < 0.001 and ^****^p < 0.0001 vs. the control group; NS, non-significant different compared to the hemin group. ^&⁣&^*p* < 0.01 and ^&⁣&⁣&^*p* < 0.001 vs. the hemin group.

Firstly, to ascertain the optimal concentration and time for the toxic effect of hemin treatment on PC12 cells, cells were treated with different concentrations of hemin for 3, 6, 12, and 24 h, and the cell viability was evaluated by CCK-8. The results showed that the cell survival rate was declined in a concentration-dependent manner with the prolongation of hemin treatment time (Figure 4A). Then, we selected 50 μM as the optimal dose for hemin treatment, and treated the PC12 cells for 12 h, and then, we used the same dose in subsequent experiments.

Secondly, to determine the optimal concentration of OXA to protect PC12 cells against hemin-induced neurotoxicity, cells were pre-treated with 10, 100, and 1,000 nM OXA for 6 h and then incubated with 50 μM hemin for 12 h, and the cell viability was evaluated by CCK-8. The results demonstrated that the cell survival rates were significantly higher in the 10, 100, and 1,000 nM pre-treatment groups than in the group treated with hemin alone (Figure 4B). Moreover, cell viability in the group pre-treated with 100 nM OXA was almost as high as that in the group pre-treated with 1,000 nM OXA (Figure 4B). Based on these results, treatment with 100 nM OXA for 6 h was considered optimal and was performed in subsequent experiments.

Thirdly, to confirm the neuroprotective effect of OXA *in vitro*, PC12 cells were pre-treated with 100 nM OXA for 6 h and then 50 μM hemin was added for 12 h. Subsequently, PC12 cells viability was evaluated using the CCK-8 assay, and cell apoptosis was analyzed by the flow cytometry method. The results demonstrated that the apoptosis ratio was significantly increased in the hemin group compared with the control group. In contrast, the apoptosis rate was significantly lower in the OXA group than in the group treated with hemin (Figures 4D, E). In line with the flow cytometry results, CCK-8 assays also observed the same trend (Figure 4C). These results suggest that OXA relieves hemin-induced injury of PC12 cells *in vitro*.

#### 3.4.2 Hemin-induced injury triggers autophagy, and OXA inhibits autophagy

**FIGURE 5:**
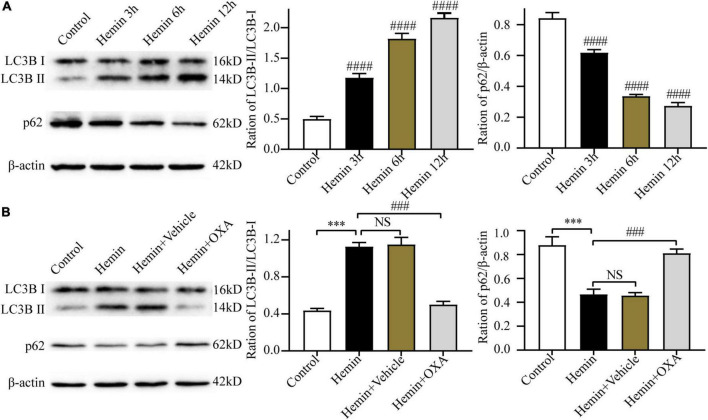
Hemin-induced injury triggers autophagy, and OXA inhibits autophagy. **(A)** The expression changes of autophagy-related proteins (LC3B and p62) at different time points after hemin-induced injury. **(B)** Western blot images of LC3B and p62 and results of quantitative analysis in each group of PC12 cells. ^***^p < 0.001 and ^####^*p* < 0.0001 vs. the control group; NS, non-significant different compared to the control group. ^###^*p* < 0.001 vs. the hemin group.

To ascertain the above results *in vitro*, we further investigated the expression levels of LC3B and p62 in PC12 cells at 3, 6, and 12 h after hemin treatment. The results of Western blotting showed that with an increase in the hemin treatment time, the ratio of LC3B-II/LC3B-I was increased and the content of p62 was progressively decreased, showing a time-dependent effect (Figure 5A).

To observe the role of OXA in autophagy in an *in vitro* model of ICH, the Western blotting was used to determine the ratio of LC3B-II/LC3B-I and the p62 level. The results showed that compared with the control group, the ratio of LC3B-II/LC3B-I were significantly elevated and the level of p62 was decreased after hemin-induced injury, whereas the OXA-treated group obtained the opposite results (Figure 5B).

#### 3.4.3 Rapamycin stimulates autophagy and reversed the protective effect of OXA

**FIGURE 6:**
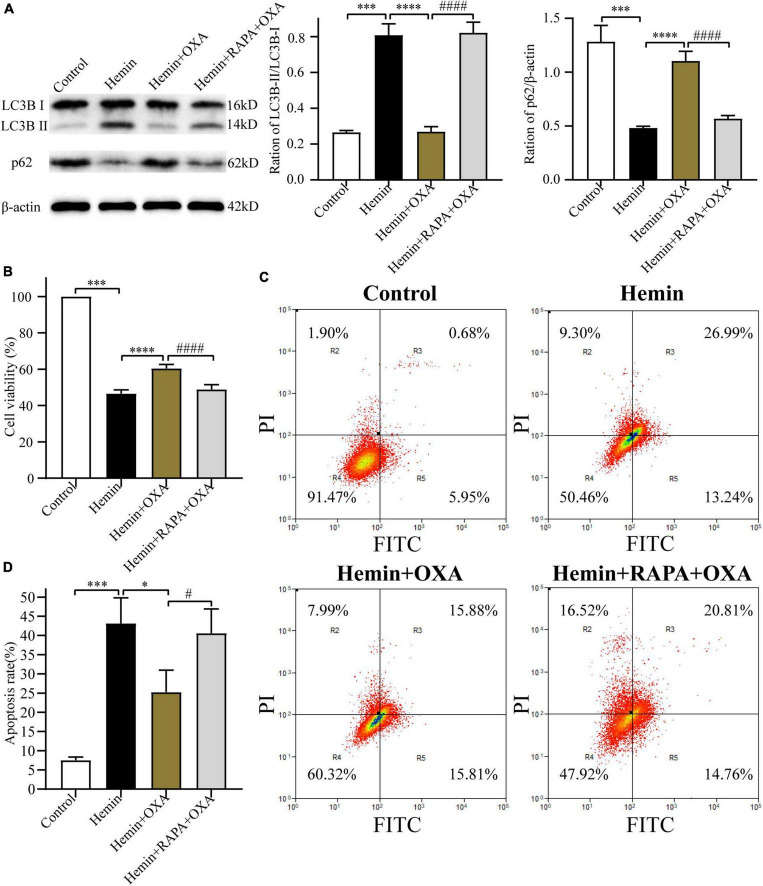
Rapamycin stimulates autophagy and reverses the neuroprotective effect of OXA. **(A)** Western blot images of LC3B and p62 and results of quantitative analysis in each group. **(B)** OXA attenuates the hemin-induced decrease in cell viability, while aggravated it after rapamycin administration. **(C)** Representative FCM plots of Annexin V-FITC apoptosis detection assay showed that autophagy activator increased apoptosis rate, and reversed the neuroprotective effect of OXA. **(D)** Quantified results of the flow cytometric analysis. ^***^p < 0.001 vs. the control group; *p < 0.05, ^****^p < 0.0001 vs. the hemin group; ^#^*p* < 0.05, ^####^*p* < 0.0001 vs. the hemin + OXA group.

To further investigate the relationship between autophagy and the protective effect of OXA, PC12 cells were pretreated with rapamycin (an allosteric inhibitor of the mammalian target of rapamycin complex 1; Cayman Chemical; Ann Arbor, MI, USA) before the addition of OXA. The autophagy-related protein expression was detected by Western blotting. OXA could significantly increase the expression levels of p62, and decrease the ratio of LC3B-II/LC3B-I, while partly blocked with rapamycin pretreatment (Figure 6A). The CCK-8 assay demonstrated that pretreated with rapamycin could decrease the cell viability of PC12, compared with OXA treatment alone (Figure 6B). Flow cytometry for apoptosis showed similar results (Figures 6C, D). In summary, OXA may exert a protective effect by regulating autophagy activity.

#### 3.4.4 OXA inhibits autophagy *via* ERK and mTOR signaling

**FIGURE 7:**
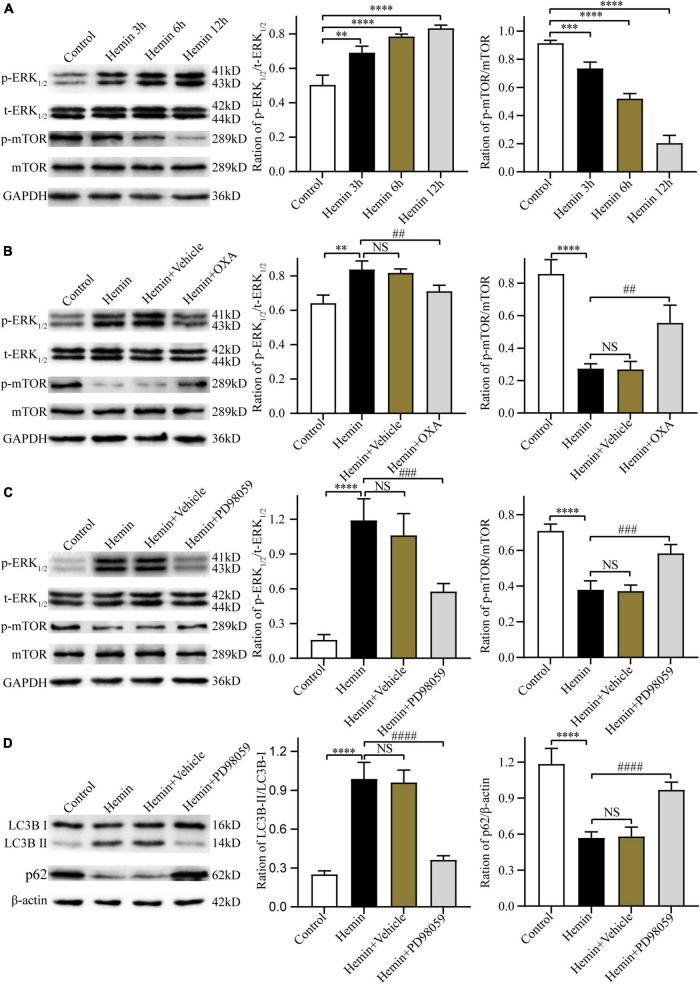
The neuroprotective effects of OXA are mediated *via* the ERK/mTOR signaling pathway. **(A)** Representative western blots of p-mTOR and p-ERK_1/2_ and results of quantitative analysis in PC12 cells treated with Hemin for 3, 6, and 12 h. **(B)** Western blot images of p-mTOR and p-ERK_1/2_ and results of quantitative analysis in each group. **(C)** Representative western blots of p-mTOR and p-ERK_1/2_ and results of quantitative analysis in PC12 cells treated with Hemin and PD98059. **(D)** Western blot images of LC3B and p62 and results of quantitative analysis in each group. ^**^p < 0.01, ^***^p < 0.001 and ^****^p < 0.0001 vs. the control group; NS, non-significant different compared to the hemin group. ^##^*p* < 0.01, ^###^*p* < 0.001 and ^####^*p* < 0.0001 vs. the hemin group.

Kim et al. (2014) and Laplante and Sabatini (2012) have reported that the ERK and mTOR signaling pathway can regulate autophagy. Thus, the present research investigated whether OXA exerts neuroprotective effects through the ERK/mTOR signaling pathway. Firstly, after treatment with hemin and OXA, the protein expression levels of p-ERK_1/2_/t-ERK_1/2_/p-mTOR were detected by Western blotting. The results showed that the level of p-mTOR protein was decreased with the aggravation of hemin-induced injury, while the level of p-ERK_1/2_ was increased (Figure 7A). As shown in Figure 7B, relative to the hemin treatment group, the expression of p-ERK_1/2_ remarkably downregulated after OXA treatment, whereas the expression of p-mTOR remarkably upregulated.

Next, we used PD98059, a non-competitive inhibitor of MAPK kinase or MEK kinase, to ascertain whether ERK is a critical factor of the upstream pathway of autophagy in hemin-induced injury. The results demonstrated that, relative to cells treated with hemin alone, pre-treated with PD98059 remarkably downregulated the expression of p-ERK_1/2_ and upregulated the expression of p-mTOR (Figure 7C). Meanwhile, PD98059 significantly downregulated the LC3B-II/LC3B-I ratio and upregulated the expression of p62 (Figure 7D). These results indicate that OXA plays a protective role by inhibiting autophagy *via* the ERK/mTOR signaling pathway.

### 3.5 OXA coupled with OXR1 protects against hemin-induced PC12 cells injury

**FIGURE 8:**
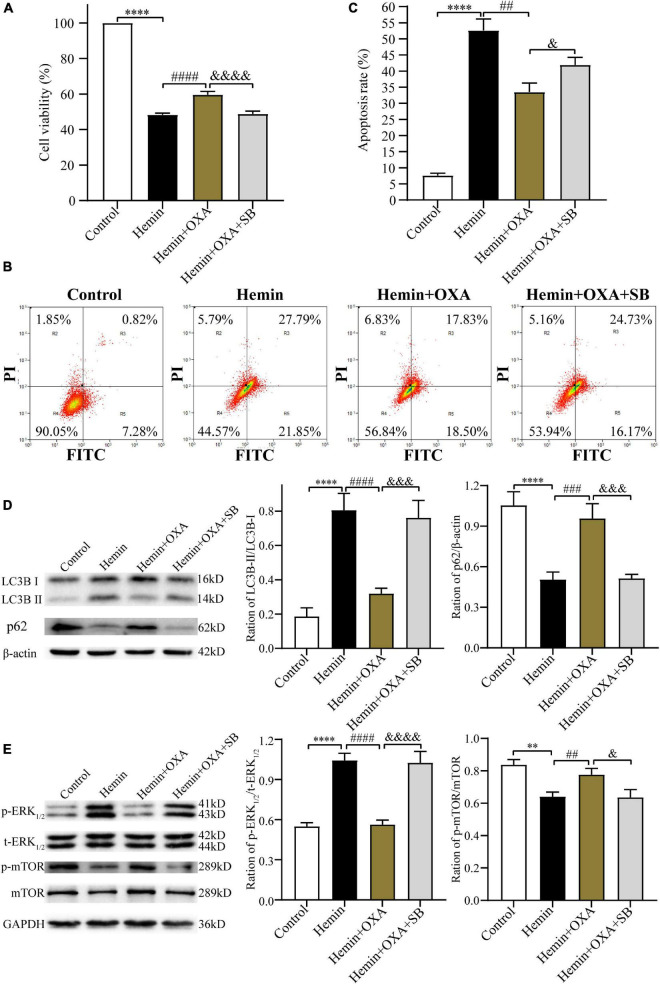
The protective effect of OXA needs to be combined with OXR1. **(A)** OXA attenuates the hemin-induced decrease in cell viability, while aggravated it after SB334867 administration. **(B)** Representative FCM plots of Annexin V-FITC apoptosis detection assay showed that OXR1 inhibitor increased apoptosis rate, and reversed the neuroprotective effect of OXA. **(C)** Quantified results of the flow cytometric analysis. **(D)** Western blot images of LC3B and p62 and results of quantitative analysis in each group. **(E)** Representative western blots of p-mTOR and p-ERK_1/2_ and results of quantitative analysis in PC12 cells treated with OXA and SB334867. ^**^p < 0.01 and ^****^p < 0.0001 vs. the control group; ^##^*p* < 0.01, ^###^*p* < 0.001 and ^####^*p* < 0.0001 vs. the hemin group; ^&^*p* < 0.05, ^&⁣&⁣&^*p* < 0.001 and ^&⁣&⁣&⁣&^*p* < 0.0001 vs. the hemin + OXA group.

The above findings have confirmed the protective effect and possible signaling pathway of OXA following ICH injury. In subsequent research, to further elucidate the neuroprotective of OXA, we pre-treated PC12 cells with the OXR1 antagonist SB334867. The CCK-8 assay was used to measure cell viability, and flow cytometry was used to detect apoptosis. The findings demonstrated that relative to the control group, the cell survival rate was remarkably decreased after hemin-induced injury. However, cell viability was significantly increased in the OXA group, relative to the hemin-treated group. When hemin-induced injury cells were subsequently treated with the combination of SB334867 and OXA, cell viability was significantly reduced compared with OXA treatment alone (Figure 8A). As expected, our apoptosis measurements were consistent with the results of cells viability. The apoptotic frequencies were 49.64% in the hemin-induced ICH models. After OXA treatment, the apoptosis rate was decreased to 36.33%. Likewise, following treatment with SB334867 and OXA, the apoptosis rate was increased by 4.57% compared with that following OXA treatment alone (Figures 8B, C). Western blotting results demonstrated that the ratio of LC3B-II/LC3B-I and the level of p-ERK_1/2_ was significantly downregulated, whereas the levels of p-mTOR and p62 were remarkably increased with OXA treatment (Figures 8D, E). The above functions of OXA were remarkably reversed by OXR1 antagonist (SB334867). These results confirmed that the neuroprotective role of OXA is dependent on the combination with OXR1.

## 4 Discussion

In this research, we attempted to elucidate the therapeutic potential of orexin A (OXA) in alleviating SBI following ICH. Our findings suggested that OXA has neuroprotective effects and can reduce EBI and neuronal death after ICH. Specifically, we observed that: (i) OXA improved neurological dysfunction and mitigated brain damage in a rat ICH model; (ii) OXA alleviated apoptosis in a hemin-induced injury model of PC12 cells; (iii) OXA inhibits activation of autophagy following ICH by binding to the OXR1 receptor; and (iv) the anti-autophagy roles of OXA may be related to the ERK/mTOR pathway.

Recent research has shown that the change in orexins and their receptors might be related to stroke incident. Reduced cerebrospinal fluid (CSF) OXA levels have been reported to occur in patients with SAH and ICH (Dohi et al., 2005, 2008). Based on these results, it can be concluded that the orexin system may play a critical role in the pathophysiology of ICH. Likewise, a continuous decline of OXA level in CSF and a rise of expression of OXR1 in the cortical region was discovered in patients with cerebral ischemia (Nakamachi et al., 2005), suggesting an involvement of the orexin system in an ischemic insult. Recently, the neuroprotective of OXA in nervous system disorders, especially in cerebrovascular diseases, have been verified. Exogenous OXA injection effectively reduced the range of infarction and significantly improved the neurological function in a CIRI model in mouse and rats (Kitamura et al., 2010; Harada et al., 2011). Xu et al. (2021) proved that OXA alleviates damage induced by oxygen–glucose deprivation/reoxygenation (OGD/R) or MCAO by inhibiting autophagy. Likewise, Yuan et al. (2011) confirmed that OXA injection remarkably improved the motor and cognitive functions of rats with CIRI. On the basis of these research, we hypothesized that OXA might have a similar neuroprotective role in ICH rats. By means of neurobehavioral tests including mNSS detection and corner turn test at 48 h following ICH, combined with determination of BWC, we ascertained that OXA could dramatically ameliorate the short-term neurological function. H&E and Nissl staining showed that treatment with OXA alleviated neuronal damage after ICH. Hemin-induced injury in PC12 cells were assessed using the CCK-8 assay and flow cytometry method. We determined the optimal dosage of OXA for the subsequent research studies and further confirmed that OXA relieves hemin-induced injury in PC12 cells *in vitro*.

Autophagy is a highly conserved cellular physiological process, in which misfolded or damaged proteins are isolated in double membrane structure referred to as autophagosomes, delivered to lysosomes, and digested by lysosomal hydrolases. It is a major pathway to remove senescent or damaged organelles and long-lived proteins in eukaryotic cells, and it plays a critical role in maintaining intracellular homeostasis and recycling cellular components. Recently research have been confirmed that autophagy is associated with many CNS disorders, including cerebral ischemia (Xu et al., 2021), trauma (Cui et al., 2015), SAH (Jiang et al., 2021), and ICH (Yuan et al., 2017). In our study, we confirmed that autophagy occurs in the brain after ICH. Results of Western blotting demonstrated that the ratio of LC3B-II/LC3B-I following ICH was dramatically increased at 12 h, and it peaked at day 2 and decreased remarkably at day 14. In contrast, the protein expression level of p62 showed an opposite trend at the same time points, which indicated stimulation of the autophagy response. Similar results were obtained in *in vitro* experiments. The results in these *in vitro* and *in vivo* ICH models are consistent with the observations of other laboratories (He et al., 2008; Chen et al., 2012), which further demonstrate that autophagy can be activated following ICH.

Apoptosis is one of the main programmed cell death pathways leading to secondary cell death around hematoma after ICH. Inhibition of apoptosis can reduce brain edema and tissue damage and improve functional prognosis after experimental ICH (Krafft et al., 2012). In addition, a programmed form of cell death has been identified and named as autophagic cell death, also known as type II programmed cell death (Ouyang et al., 2012). Recent studies have suggested that autophagic cell death occurs after ICH and inhibition of autophagy contributes to alleviate iron-induced brain injury (Chen et al., 2012). A clinical study has shown that the number of autophagic neurons in patients with ICH is related to the severity of neuronal dysfunction and blood volume (Wu et al., 2019). These data suggest autophagy plays a detrimental role in ICH. In our *in vivo* and *in vitro* experiments, by immunohistochemistry and Western blotting, we observed that OXA can inhibit the activation of autophagy. We confirmed the immunohistochemistry and Western blotting findings by the most sensitive and accurate transmission electron microscopy that is for determining whether cells are undergoing autophagy. When PC12 cells were co-treated with the autophagy activator rapamycin and OXA, we found that the neuroprotective roles of OXA were weakened. Thus, we presumed that the neuroprotective role of OXA may be achieved by suppressing autophagy.

Autophagy is accurately controlled by a variety of signaling pathways, such as PI3K/AKT/mTOR (Vergadi et al., 2017; Li W. D. et al., 2018), Ras/Raf/MEK/ERK_1/2_ etc. (Kyriakakis et al., 2017; Yiu et al., 2018). The PI3K/AKT pathway is the classical autophagy pathway. Meanwhile, mTOR, as a downstream effector of the PI3K (phosphatidylinositol 3-kinase)/PKB (protein kinase B) signaling pathway, also exerts a significant role in CNS apoptosis and autophagy (Wu et al., 2018). Multiple cellular pathophysiological processes, including autophagy, are regulated by extracellular signal-regulated kinase (ERK) (Wen et al., 2016). An enhanced activity of ERK_1/2_ has been reported to be closely related to the control of autophagy activation. Activation of the ERK_1/2_ signal pathway has also been reported to increase the expression of autophagy-related genes and proteins. To explore whether OXA could inhibit PC12 cells autophagy *via* mTOR/ERK_1/2_ signaling pathway *in vitro*, we analyzed p-mTOR and p-ERK_1/2_ expression after hemin-induced injury. We found that with aggravation of hemin-induced injury, the expression of p-ERK_1/2_ was gradually increased, but the mTOR level was gradually decreased. However, the expression of p-ERK_1/2_ was dramatically decreased and the expression of p-mTOR was significantly increased after OXA treatment. Then, we treated PC12 cells with hemin-induced injury using an ERK specific antagonist PD98059. We found that the ERK inhibitor remarkably suppressed the p-ERK_1/2_ and LC3B-II/LC3B-I expression levels, but it increased p-mTOR expression in PC12 cells after hemin-induced injury. These data indicated that OXA may inhibit autophagy to play neuroprotective effect through the ERK and mTOR pathway.

It has been reported that OXR1 and OXR2 might exert different effects. Scammell and Winrow (2011) demonstrated that OXR2 or non-competitive orexins inhibitors were effective in treating insomnia, suggesting that OXR2 may play a more important role. Irving observed an increase in the OXR1 gene expression in rats with cerebral ischemia (Irving et al., 2002), while OXR2 gene expression did not change significantly. In this present research, we found that the neuroprotective role of OXA was remarkably reversed by SB-334867. Therefore, we conclude that the role of these two receptors is different; OXR1 is more participated in the ERK/mTOR signaling pathway and mediates the neuroprotective role following ICH.

After ICH, nerve cells suffer secondary injury caused by various pathological processes, such as inflammation, autophagy, and oxidative stress (Zhang and Liu, 2020). On the basis of our results, we would like to suggest that OXA, a polypeptide that can improve neurological functional defects after ICH, is neuroprotective. In this regard, one possibility is that OXA can reduce the level of autophagy in tissues surrounding hemorrhagic lesions after ICH. There has been reported evidence indicating that OXA can significantly improve neurofunctional outcomes after ICH through alleviating neuroinflammation (Li et al., 2020). Thus, the neuroprotective effect of OXA might also be indirectly mediated to some extent by suppressing inflammasome activation or directly sequester inflammasomes *via* autophagy-lysosomal pathway. However, the mechanism of interaction between autophagy and inflammation needs to be further studied. In addition, although we have confirmed that OXR1 is more involved in the neuroprotective effects of OXA after ICH, the details of the different roles of the two receptors remain unclarified. Therefore, it will be critical to explore these mechanisms in future studies.

## 5 Conclusion

In summary, OXA exerts a neuroprotective role in ICH rats by inhibiting autophagy through the OXR1-mediated ERK/mTOR pathway, and it might provide a novel therapeutic approach in patients suffering from ICH. More studies are needed to validate our findings and provide more evidence for OXA to treat ICH in clinical practice.

## Data availability statement

The original contributions presented in this study are included in the article/supplementary material, further inquiries can be directed to the corresponding author.

## Ethics statement

This animal study was reviewed and approved by the Laboratory Animal Ethical and Welfare Committee of North China University of Science and Technology.

## Author contributions

JC was responsible for experimental design and revised the manuscript. DZ performed the experiments and wrote the manuscript. MZ and JW carried out the experiments and analyzed the data. YC, CL, and XZ prepared the figures. KW provided some technical supports. All authors contributed to the article and approved the submitted version.

## References

[B1] Ahmadi-Soleimani S. M. Mianbandi V. Azizi H. Azhdari-Zarmehri H. Ghaemi-Jandabi M. Abbasi-Mazar A. (2020). Coregulation of sleep-pain physiological interplay by orexin system: An unprecedented review. *Behav. Brain Res.* 391:112650. 10.1016/j.bbr.2020.112650 32454053

[B2] Appelboom G. Hwang B. Y. Bruce S. S. Piazza M. A. Kellner C. P. Meyers P. M. (2012). Predicting outcome after arteriovenous malformation-associated intracerebral hemorrhage with the original ICH score. *World Neurosurg.* 78 646–650. 10.1016/j.wneu.2011.12.001 22381312

[B3] Barinaga M. (1998). New appetite-boosting peptides found. *Science* 279:1134. 10.1126/science.279.5354.1134 9508686

[B4] Benjamin E. J. Blaha M. J. Chiuve S. E. Cushman M. Das S. R. Deo R. (2017). Heart disease and stroke statistics-2017 update: A report from the American Heart Association. *Circulation* 135 e146–e603. 10.1161/CIR.0000000000000485 28122885 PMC5408160

[B5] Bennett H. L. Fleming J. T. O’Prey J. Ryan K. M. Leung H. Y. (2010). Androgens modulate autophagy and cell death via regulation of the endoplasmic reticulum chaperone glucose-regulated protein 78/BiP in prostate cancer cells. *Cell Death Dis.* 1:e72. 10.1038/cddis.2010.50 21364676 PMC3032338

[B6] Boya P. Reggiori F. Codogno P. (2013). Emerging regulation and functions of autophagy. *Nat. Cell Biol.* 15 713–720. 10.1038/ncb2788 23817233 PMC7097732

[B7] Carloni S. Buonocore G. Balduini W. (2008). Protective role of autophagy in neonatal hypoxia-ischemia induced brain injury. *Neurobiol. Dis.* 32 329–339. 10.1016/j.nbd.2008.07.022 18760364

[B8] Chen C.-W. Chen T.-Y. Tsai K.-L. Lin C.-L. Yokoyama K. K. Lee W.-S. (2012). Inhibition of autophagy as a therapeutic strategy of iron-induced brain injury after hemorrhage. *Autophagy* 8 1510–1520. 10.4161/auto.21289 22909970

[B9] Cui C. M. Gao J. L. Cui Y. Sun L. Q. Wang Y. C. Wang K. J. (2015). Chloroquine exerts neuroprotection following traumatic brain injury via suppression of inflammation and neuronal autophagic death. *Mol. Med. Rep.* 12 2323–2328. 10.3892/mmr.2015.3611 25872478

[B10] Dang G. Yang Y. Wu G. Hua Y. Keep R. F. Xi G. (2017). Early erythrolysis in the hematoma after experimental intracerebral hemorrhage. *Transl. Stroke Res.* 8 174–182. 10.1007/s12975-016-0505-3 27783383 PMC5350065

[B11] Dohi K. Ripley B. Fujiki N. Ohtaki H. Shioda S. Aruga T. (2005). CSF hypocretin-1/orexin-A concentrations in patients with subarachnoid hemorrhage (SAH). *Peptides* 26 2339–2343. 10.1016/j.peptides.2005.04.004 15893406

[B12] Dohi K. Ripley B. Fujiki N. Ohtaki H. Yamamoto T. Goto Y. (2008). CSF orexin-A/hypocretin-1 concentrations in patients with intracerebral hemorrhage (ICH). *Regul. Pept.* 145 60–64. 10.1016/j.regpep.2007.08.005 17868933

[B13] Drouot X. Moutereau S. Nguyen J. P. Lefaucheur J. P. Créange A. Remy P. (2003). Low levels of ventricular CSF orexin/hypocretin in advanced PD. *Neurology* 61 540–543. 10.1212/01.wnl.0000078194.53210.48 12939433

[B14] Duan X. C. Wang W. Feng D. X. Yin J. Zuo G. Chen D. D. (2017). Roles of autophagy and endoplasmic reticulum stress in intracerebral hemorrhage-induced secondary brain injury in rats. *CNS Neurosci. Ther.* 23 554–566. 10.1111/cns.12703 28544790 PMC6492729

[B15] Feng Y. Liu T. Li X. Q. Liu Y. Zhu X. Y. Jankovic J. (2014). Neuroprotection by Orexin-A via HIF-1α induction in a cellular model of Parkinson’s disease. *Neurosci. Lett.* 579 35–40. 10.1016/j.neulet.2014.07.014 25038418

[B16] Füllgrabe J. Klionsky D. J. Joseph B. (2013). Histone post-translational modifications regulate autophagy flux and outcome. *Autophagy* 9 1621–1623. 10.4161/auto.25803 23934085 PMC5860799

[B17] Gan-Or Z. Dion P. A. Rouleau G. A. (2015). Genetic perspective on the role of the autophagy-lysosome pathway in Parkinson disease. *Autophagy* 11 1443–1457. 10.1080/15548627.2015.1067364 26207393 PMC4590678

[B18] Harada S. Fujita-Hamabe W. Tokuyama S. (2011). Effect of orexin-A on post-ischemic glucose intolerance and neuronal damage. *J. Pharmacol. Sci.* 115 155–163. 10.1254/jphs.10264FP 21258173

[B19] He Y. Wan S. Hua Y. Keep R. F. Xi G. (2008). Autophagy after experimental intracerebral hemorrhage. *J. Cereb. Blood Flow Metab.* 28 897–905. 10.1038/sj.jcbfm.9600578 17987045

[B20] Hu X. Tao C. Gan Q. Zheng J. Li H. You C. (2016). Oxidative stress in intracerebral hemorrhage: Sources, mechanisms, and therapeutic targets. *Oxid. Med. Cell Longev.* 2016:3215391. 10.1155/2016/3215391 26843907 PMC4710930

[B21] Irving E. A. Harrison D. C. Babbs A. J. Mayes A. C. Campbell C. A. Hunter A. J. (2002). Increased cortical expression of the orexin-1 receptor following permanent middle cerebral artery occlusion in the rat. *Neurosci. Lett.* 324 53–56. 10.1016/s0304-3940(02)00176-3 11983293

[B22] Jiang B. Li Y. Dai W. Wu A. Wu H. Mao D. (2021). Hydrogen-rich saline alleviates early brain injury through regulating of ER stress and autophagy after experimental subarachnoid hemorrhage. *Acta Cir. Bras.* 36:e360804. 10.1590/ACB360804 34644772 PMC8516430

[B23] Jing C. h. Wang L. Liu P. p. Wu C. Ruan D. Chen G. (2012). Autophagy activation is associated with neuroprotection against apoptosis via a mitochondrial pathway in a rat model of subarachnoid hemorrhage. *Neuroscience* 213 144–153. 10.1016/j.neuroscience.2012.03.055 22521819

[B24] Kang M. Yao Y. (2019). Oligodendrocytes in intracerebral hemorrhage. *CNS Neurosci. Therap.* 25 1075–1084. 10.1111/cns.13193 31410988 PMC6776757

[B25] Kim J. H. Hong S. K. Wu P. K. Richards A. L. Jackson W. T. Park J. I. (2014). Raf/MEK/ERK can regulate cellular levels of LC3B and SQSTM1/p62 at expression levels. *Exp. Cell Res.* 327 340–352. 10.1016/j.yexcr.2014.08.001 25128814 PMC4164593

[B26] Kim Y. J. Baek E. Lee J. S. Lee G. M. (2013). Autophagy and its implication in Chinese hamster ovary cell culture. *Biotechnol. Lett.* 35 1753–1763. 10.1007/s10529-013-1276-5 23881315

[B27] Kim-Han J. S. Kopp S. J. Dugan L. L. Diringer M. N. (2006). Perihematomal mitochondrial dysfunction after intracerebral hemorrhage. *Stroke* 37 2457–2462. 10.1161/01.STR.0000240674.99945.4e 16960094

[B28] Kitamura E. Hamada J. Kanazawa N. Yonekura J. Masuda R. Sakai F. (2010). The effect of orexin-A on the pathological mechanism in the rat focal cerebral ischemia. *Neurosci. Res.* 68 154–157. 10.1016/j.neures.2010.06.010 20600373

[B29] Kotoda M. Furukawa H. Miyamoto T. Korai M. Shikata F. Kuwabara A. (2018). Role of myeloid lineage cell autophagy in ischemic brain injury. *Stroke* 49 1488–1495. 10.1161/strokeaha.117.018637 29748423 PMC5970995

[B30] Krafft P. R. Altay O. Rolland W. B. Duris K. Lekic T. Tang J. (2012). α7 nicotinic acetylcholine receptor agonism confers neuroprotection through GSK-3β inhibition in a mouse model of intracerebral hemorrhage. *Stroke* 43 844–850. 10.1161/strokeaha.111.639989 22207510 PMC3293395

[B31] Kyriakakis E. Frismantiene A. Dasen B. Pfaff D. Rivero O. Lesch K. P. (2017). T-cadherin promotes autophagy and survival in vascular smooth muscle cells through MEK1/2/Erk1/2 axis activation. *Cell Signal.* 35 163–175. 10.1016/j.cellsig.2017.04.004 28392425

[B32] Laplante M. Sabatini D. M. (2012). mTOR signaling in growth control and disease. *Cell* 149 274–293. 10.1016/j.cell.2012.03.017 22500797 PMC3331679

[B33] Li H. Wu J. Shen H. Yao X. Liu C. Pianta S. (2018). Autophagy in hemorrhagic stroke: Mechanisms and clinical implications. *Prog. Neurobiol.* 163–164 79–97. 10.1016/j.pneurobio.2017.04.002 28414101

[B34] Li T. Xu W. Ouyang J. Lu X. Sherchan P. Lenahan C. (2020). Orexin A alleviates neuroinflammation via OXR2/CaMKKbeta/AMPK signaling pathway after ICH in mice. *J. Neuroinflammation* 17:187. 10.1186/s12974-020-01841-1 32539736 PMC7294616

[B35] Li W. D. Zhou D. M. Sun L. L. Xiao L. Liu Z. Zhou M. (2018). LncRNA WTAPP1 promotes migration and angiogenesis of endothelial progenitor cells via MMP1 through MicroRNA 3120 and Akt/PI3K/autophagy pathways. *Stem Cells* 36 1863–1874. 10.1002/stem.2904 30171660

[B36] Manaenko A. Fathali N. Khatibi N. H. Lekic T. Hasegawa Y. Martin R. (2011). Arginine-vasopressin V1a receptor inhibition improves neurologic outcomes following an intracerebral hemorrhagic brain injury. *Neurochem. Int.* 58 542–548. 10.1016/j.neuint.2011.01.018 21256175 PMC3063401

[B37] Mohammad Ahmadi Soleimani S. Azizi H. Mirnajafi-Zadeh J. Semnanian S. (2015). Orexin type 1 receptor antagonism in rat locus coeruleus prevents the analgesic effect of intra-LC met-enkephalin microinjection. *Pharmacol. Biochem. Behav.* 136 102–106. 10.1016/j.pbb.2015.07.010 26210888

[B38] Mracsko E. Veltkamp R. (2014). Neuroinflammation after intracerebral hemorrhage. *Front. Cell Neurosci.* 8:388. 10.3389/fncel.2014.00388 25477782 PMC4238323

[B39] Nakamachi T. Endo S. Ohtaki H. Yin L. Kenji D. Kudo Y. (2005). Orexin-1 receptor expression after global ischemia in mice. *Regul. Pept.* 126 49–54. 10.1016/j.regpep.2004.08.037 15620413

[B40] Ning J. Junyi T. Chang M. Yueting W. Jingyan Z. Jin Z. (2019). TOM7 silencing exacerbates focal cerebral ischemia injury in rat by targeting PINK1/Beclin1-mediated autophagy. *Behav. Brain Res.* 360 113–119. 10.1016/j.bbr.2018.11.031 30468788

[B41] Ouyang L. Shi Z. Zhao S. Wang F. T. Zhou T. T. Liu B. (2012). Programmed cell death pathways in cancer: A review of apoptosis, autophagy and programmed necrosis. *Cell Prolif.* 45 487–498. 10.1111/j.1365-2184.2012.00845.x 23030059 PMC6496669

[B42] Rami A. Langhagen A. Steiger S. (2008). Focal cerebral ischemia induces upregulation of Beclin 1 and autophagy-like cell death. *Neurobiol. Dis.* 29 132–141. 10.1016/j.nbd.2007.08.005 17936001

[B43] Salminen A. Kaarniranta K. Kauppinen A. Ojala J. Haapasalo A. Soininen H. (2013). Impaired autophagy and APP processing in Alzheimer’s disease: The potential role of Beclin 1 interactome. *Prog. Neurobiol.* 10 33–54. 10.1016/j.pneurobio.2013.06.002 23827971

[B44] Scammell T. E. Winrow C. J. (2011). Orexin receptors: Pharmacology and therapeutic opportunities. *Annu. Rev. Pharmacol. Toxicol.* 51 243–266. 10.1146/annurev-pharmtox-010510-100528 21034217 PMC3058259

[B45] Shen X. Ma L. Dong W. Wu Q. Gao Y. Luo C. (2016). Autophagy regulates intracerebral hemorrhage induced neural damage via apoptosis and NF-κB pathway. *Neurochem. Int.* 96 100–112. 10.1016/j.neuint.2016.03.004 26964766

[B46] Vergadi E. Ieronymaki E. Lyroni K. Vaporidi K. Tsatsanis C. (2017). Akt signaling pathway in macrophage activation and M1/M2 polarization. *J. Immunol.* 198 1006–1014. 10.4049/jimmunol.1601515 28115590

[B47] Wang C. Wang Q. Ji B. Pan Y. Xu C. Cheng B. (2018). The orexin/receptor system: Molecular mechanism and therapeutic potential for neurological diseases. *Front. Mol. Neurosci.* 11:220. 10.3389/fnmol.2018.00220 30002617 PMC6031739

[B48] Wellnitz K. Taegtmeyer H. (2010). Mechanical unloading of the failing heart exposes the dynamic nature of autophagy. *Autophagy* 6 155–156. 10.4161/auto.6.1.10538 19949312

[B49] Wen J. Zhao Y. Guo L. (2016). Orexin A induces autophagy in HCT-116 human colon cancer cells through the ERK signaling pathway. *Int. J. Mol. Med.* 37 126–132. 10.3892/ijmm.2015.2409 26572581

[B50] Wirawan E. Vanden Berghe T. Lippens S. Agostinis P. Vandenabeele P. (2012). Autophagy: For better or for worse. *Cell Res.* 22 43–61. 21912435 10.1038/cr.2011.152 PMC3351915

[B51] Wu C. Yan X. Liao Y. Liao L. Huang S. Zuo Q. (2019). Increased perihematomal neuron autophagy and plasma thrombin-antithrombin levels in patients with intracerebral hemorrhage: An observational study. *Medicine (Baltimore)* 98:e17130. 10.1097/MD.0000000000017130 31574813 PMC6775380

[B52] Wu Y. Wang L. Hu K. Yu C. Zhu Y. Zhang S. (2018). Mechanisms and therapeutic targets of depression after intracerebral hemorrhage. *Front. Psychiatry* 9:682. 10.3389/fpsyt.2018.00682 30618863 PMC6304443

[B53] Xi T. Jin F. Zhu Y. Wang J. Tang L. Wang Y. (2018). miR-27a-3p protects against blood-brain barrier disruption and brain injury after intracerebral hemorrhage by targeting endothelial aquaporin-11. *J. Biol. Chem.* 293 20041–20050. 10.1074/jbc.RA118.001858 30337368 PMC6311503

[B54] Xu D. Kong T. Zhang S. Cheng B. Chen J. Wang C. (2021). Orexin-A protects against cerebral ischemia-reperfusion injury by inhibiting excessive autophagy through OX1R-mediated MAPK/ERK/mTOR pathway. *Cell Signal.* 79:109839. 10.1016/j.cellsig.2020.109839 33212156

[B55] Yang G. Qian C. Zhang C. Bao Y. Liu M. Y. Jiang F. (2021). Hepcidin attenuates the iron-mediated secondary neuronal injury after intracerebral hemorrhage in rats. *Transl. Res.* 229 53–68. 10.1016/j.trsl.2020.09.002 32932001

[B56] Yiu S. P. T. Hui K. F. Choi C. K. Kao R. Y. T. Ma C. W. Yang D. (2018). Intracellular iron chelation by a novel compound, C7, reactivates epstein-barr virus (EBV) lytic cycle via the ERK-autophagy axis in EBV-positive epithelial cancers. *Cancers (Basel)* 10 505. 10.3390/cancers10120505 30544928 PMC6316324

[B57] Yu S. Yu M. He X. Wen L. Bu Z. Feng J. (2019). KCNQ1OT1 promotes autophagy by regulating miR-200a/FOXO3/ATG7 pathway in cerebral ischemic stroke. *Aging Cell* 18:e12940. 10.1111/acel.12940 30945454 PMC6516167

[B58] Yuan B. Shen H. Lin L. Su T. Zhong L. Yang Z. (2017). Autophagy promotes microglia activation through beclin-1-Atg5 pathway in intracerebral hemorrhage. *Mol. Neurobiol.* 54 115–124. 10.1007/s12035-015-9642-z 26732594

[B59] Yuan L. B. Dong H. L. Zhang H. P. Zhao R. N. Gong G. Chen X. M. (2011). Neuroprotective effect of orexin-A is mediated by an increase of hypoxia-inducible factor-1 activity in rat. *Anesthesiology* 114 340–354. 10.1097/ALN.0b013e318206ff6f 21239965

[B60] Zhang Y. Liu C. (2020). Autophagy and hemorrhagic stroke. *Adv. Exp. Med. Biol* 1207 135–147. 10.1007/978-981-15-4272-5_8 32671743

[B61] Zhao H. Chen Y. Feng H. (2018). P2X7 receptor-associated programmed cell death in the pathophysiology of hemorrhagic stroke. *Curr. Neuropharmacol.* 16 1282–1295. 10.2174/1570159X16666180516094500 29766811 PMC6251042

[B62] Zhao M. Gao J. Cui C. Zhang Y. Jiang X. Cui J. (2021). Inhibition of PTEN ameliorates secondary hippocampal injury and cognitive deficits after intracerebral hemorrhage: Involvement of AKT/FoxO3a/ATG-mediated autophagy. *Oxid. Med. Cell Longev.* 2021:5472605. 10.1155/2021/5472605 33777313 PMC7969103

[B63] Zhao X. R. Gonzales N. Aronowski J. (2015). Pleiotropic role of PPARgamma in intracerebral hemorrhage: An intricate system involving Nrf2, RXR, and NF-kappaB. *CNS Neurosci. Ther.* 21 357–366. 10.1111/cns.12350 25430543 PMC4376579

[B64] Zhou Y. F. Zhang C. Yang G. Qian Z. M. Zhang M. W. Ma J. (2017). Hepcidin protects neuron from hemin-mediated injury by reducing iron. *Front. Physiol.* 8:332. 10.3389/fphys.2017.00332 28588503 PMC5440571

